# Supplementary material for: Identification of photoperiod-induced specific miRNAs in the adrenal glands of Sunite sheep (Ovis aries)
Source: Front Vet Sci. 2022 Jul 22;9:888207. doi: 10.3389/fvets.2022.888207 (PMC9354845; doi:10.3389/fvets.2022.888207)
Supplement: Supplementary file 1 [file Data_Sheet_1.ZIP › Supplementary file/miRDeep2 file.zip!/miRDeep2 file/Novel_Structure/LP42A2/Novel_294.pdf]

Provisional ID	:	NC_019475.2_29220
Score total	:	25.9
Score for star read(s)	:	3.9
Score for read counts	:	19
Score for mfe	:	1.4
Score for randfold	:	1.6
Score for cons. seed	:	
Total read count	:	49
Mature read count	:	46
Loop read count	:	0
Star read count	:	3

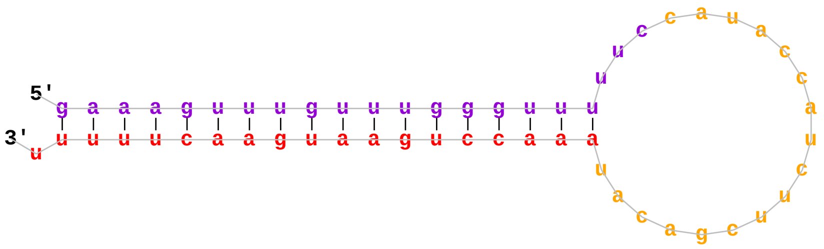

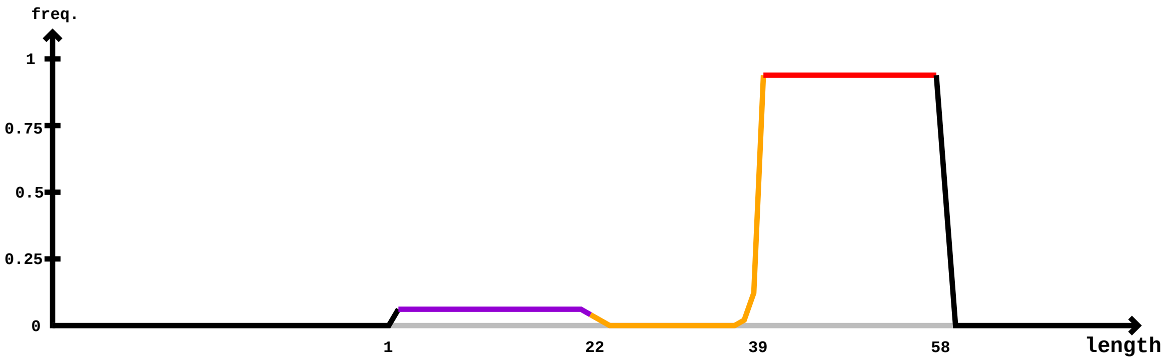

Star Mature

```
5'- cauuuacaaagugaaggcuguauugggcuggccagaaaguuuguuuggguuuuuccauaccaucuucgacauaaaccugaaugaacuuuuuagccaacccaauaaaauacu   -3'   obs
    cauuuacaaagugaaggcuguauugggcuggccagaaaguuuguuuggguuuuuccauaccaucuucgacauaaaccugaaugaacuuuuuagccaacccaauaaaauacu         exp
    .........(((....))).(((((((.((((.(((((((((((((((((((....................))))))))))))))))))).)))).)))))))......    reads   mm   sample
    ..................................gaaaguuuguuuggguuuuu.........................................................    1       0    seq
    ..................................gaaaguuuguuuggguuuuuc........................................................    2       0    seq
    ......................................................................auaaaccugaaugaacuuuuu....................    1       0    seq
    .......................................................................Caaaccugaaugaacuuuuu....................    3       1    seq
    .......................................................................Gaaaccugaaugaacuuuuu....................    1       1    seq
    .......................................................................uaaaccugaaugaacuuuuua...................    1       0    seq
    ........................................................................aaaccugaaugaacuuuuu....................    2       0    seq
    ........................................................................aaaccCgaaugaacuuuuu....................    28      1    seq
    ........................................................................aaaccCgaaugaacuuuuua...................    10      1    seq
```
